# Patient, carer and healthcare professional perspectives on deprescribing in surgical wards: A mixed methods study

**DOI:** 10.1002/bcp.70088

**Published:** 2025-05-12

**Authors:** Bonnie M. Liu, Janani Thillainadesan, Aili Langford, Kenji Fujita, Danijela Gnjidic, Sarah N. Hilmer

**Affiliations:** ^1^ Ageing and Pharmacology Laboratory, Kolling Institute, Faculty of Medicine and Health The University of Sydney and the Northern Sydney Local Health District Sydney Australia; ^2^ Aged Care Department Royal North Shore Hospital Sydney Australia; ^3^ Department of Geriatric Medicine and Centre for Education and Research on Ageing Concord Hospital Sydney Australia; ^4^ Concord Clinical School, Faculty of Medicine and Health The University of Sydney Sydney Australia; ^5^ Sydney Pharmacy School, Faculty of Medicine and Health The University of Sydney Sydney Australia; ^6^ Centre for Medicine Use and Safety, Faculty of Pharmacy and Pharmaceutical Sciences Monash University Melbourne Australia; ^7^ Clinical Pharmacology Department Royal North Shore Hospital Sydney Australia

**Keywords:** elderly, medication safety, prescribing, surgery

## Abstract

**Aims:**

The perspectives of patients and healthcare professionals regarding deprescribing in surgical wards within hospital settings are unknown. The aim of this study was to explore current practices, attitudes and the enablers and barriers to deprescribing in hospital for older surgical inpatients from the perspectives of doctors, pharmacists, patients and carers.

**Methods:**

A mixed methods study was performed. Two surveys were administered Australia‐wide (revised Patients' Attitudes Towards Deprescribing questionnaire for patients/carers and Deprescribing Self‐Efficacy Survey for doctors/pharmacists). Interviews, focus groups and observations of ward rounds were conducted with participants from five Australian hospitals. Quantitative data were analysed descriptively, while qualitative data were examined using a combined inductive and deductive approach, with results triangulated.

**Results:**

There were 109 survey participants (58 doctors/pharmacists and 51 patients/carers), 28 interview/focus group participants (15 doctors/pharmacists and 13 patients/carers) and eight ward round participants. Doctors and pharmacists reported low to moderate levels of confidence in deprescribing. While most patients and carers were satisfied with their medications, they expressed a willingness to consider deprescribing. Five themes were identified from the interviews, focus groups and ward round observations: (1) deprescribing is not a priority, (2) medication review occurs in response to triggers, (3) knowledge about deprescribing is limited, (4) deprescribing requires a team effort and (5) trust, rapport and communication are essential for successful deprescribing.

**Conclusions:**

Doctors working on surgical wards are unlikely to proactively deprescribe medications. A collaborative patient‐centred approach involving geriatricians, clinical pharmacologists and pharmacists, along with educational interventions may facilitate deprescribing for surgical patients.

**What is already known about this subject**

- Polypharmacy is common in older adults undergoing surgery and is associated with adverse outcomes.
- Comprehensive geriatric assessment, which includes a medication review, improves outcomes for older surgical inpatients.
- Previous studies have not explored the views of doctors and pharmacists working in inpatient surgical settings on deprescribing.

**What this study adds**

- Surgical doctors are unlikely to proactively review and deprescribe medications.
- Knowledge of deprescribing is limited and there is a need for further education for healthcare professionals working in surgical settings.
- A collaborative patient‐centred approach with involvement from geriatricians, clinical pharmacologists, other physicians and/or pharmacists may improve prescribing.

## 1 INTRODUCTION

Most older patients (>65 years) undergoing surgery have polypharmacy and this is associated with increased mortality and a higher rate of adverse events.^1^ Deprescribing is the process of withdrawal of an inappropriate medication, supervised by a healthcare professional with the goal of managing polypharmacy and improving outcomes.^2^ Deprescribing interventions have been shown to reduce potentially inappropriate medications, potential prescription omissions, adverse drug reactions and hospital readmissions.^3^, ^4^

Previous studies have explored patient attitudes to deprescribing in the community and in hospital, although there are limited studies investigating patients undergoing surgery. A systematic review of results from the Patients' Attitudes Towards Deprescribing questionnaire studies suggested that most patients (84%) were willing to consider deprescribing.^5^ Factors that impacted the perceived value of a medication from patient and carers' perspectives include perceived effectiveness, adverse effects on quality of life, cost and a strong relationship with the prescriber.^6^ In the hospital setting, there are challenges in medication history taking, missed opportunities for deprescribing and challenges in coordinating deprescribing recommendations.^7^

There is an established role for comprehensive geriatric assessment for improving outcomes for older surgical inpatients.^8^ A previous survey conducted in the United Kingdom suggested most (85%) surgical trainees believe support from geriatric medicine was necessary, but that in the majority of cases it was inadequate and they would support closer collaboration with geriatric medicine.^9^ Medication review is one component of the comprehensive geriatric assessment. A recent study looking at the barriers and facilitators to deprescribing before surgery from patient and primary care providers' perspectives suggested similar barriers to those identified in the primary care setting and in medical inpatients.^10^ There is limited research exploring the perspectives of healthcare professionals, patients and carers on medication review and deprescribing in inpatient surgical settings.

To inform improvements in medication review and reduce preventable medication‐related harm in older surgical inpatients, it is important to understand deprescribing from the perspectives of doctors and pharmacists working in surgical settings (hereinafter referred to as surgical healthcare professionals), as well as patients and carers in inpatient surgical settings. In this study, we aim to explore the current practices, attitudes, and the enablers and barriers to deprescribing in hospital for older surgical inpatients from the perspectives of surgical healthcare professionals as well as patients and carers.

## 2 METHODS

### 2.1 Study type

This mixed‐methods study consisted of an Australia‐wide survey and focus groups, interviews and ward round observations conducted at hospital sites. The study was approved by the institutional ethics committee (2022/ETH01284).

### 2.2 Participants and recruitment

Participants were patients aged >65 years who were admitted under any surgical team within the last 2 years, their carers, surgical doctors and pharmacists. Given that new postoperative polypharmacy is common,^11^ polypharmacy prior to admission was not a prerequisite for inclusion. Survey participants were recruited from across Australia. Interview, focus group and observation participants were recruited from the five study hospitals in one metropolitan and one regional health district. Hospital A was a metropolitan tertiary hospital, Hospitals B and C were metropolitan secondary hospitals and Hospitals D and E were regional hospitals. There are at least 10 000 surgical admissions across these hospitals each year.

Emails with study information and flyers were sent to professional and consumer networks, and the surgical heads of department at the study hospitals. Participants could opt into participating by scanning the QR code on the flyer which linked to the online REDCap survey or by contacting the researcher. Paper surveys were also offered to participants by clinicians at the study hospitals and could be returned to locked boxes or by mail. Survey participants at the five study hospitals could opt in to participating in an interview, focus group or observation. Participants were able to participate in more than one component of the study if they elected to. Participants were also recruited by snowballing. The target sample size for each group (surgical healthcare professionals and patients/carers) was 50 survey respondents. The target sample size for the interview and/or focus group participants was determined when data saturation was reached and we estimated that this would be approximately 15 interview and/or focus group participants. Informed consent was obtained. Participation was voluntary and compensation was not provided for study participants.

### 2.3 Data collection

The validated revised Patients' Attitudes Towards Deprescribing (rPATD) questionnaire was completed by older surgical patients and/or their carers.^12^ This survey includes questions on the perceived medication burden, medication appropriateness, concerns about stopping of medications and involvement in medication management. Healthcare professionals completed the validated Deprescribing Self‐Efficacy Survey^13^ in which participants self‐rated their confidence (using a Likert Scale) in deprescribing under potentially impeding circumstances. The interview questions were developed and piloted by the research team. Semi‐structured interviews and focus groups were conducted by one researcher who was a geriatrician at one of the study hospitals. These focused on knowledge, practice and attitude towards polypharmacy and deprescribing; and the enablers and barriers to optimal medication management in hospital. These were recorded, transcribed and de‐identified. The surveys and interview/focus group question guides are available in Supplemental Text S1. One researcher shadowed hospital surgical teams as they completed ward rounds and collected notes on medication review and clinician discussion of medications with patients and carers.

### 2.4 Data analysis

Descriptive statistics were used to report participant characteristics and survey results. The median scores in response to each survey question were plotted on radar charts. Survey results were also illustrated using stacked bar charts. For interviews and focus groups, two researchers independently reviewed de‐identified transcripts and coded the data. A combined inductive and deductive approach was used.^14^ The interview/focus group guide was used to structure the initial coding and an iterative process of thematic analysis was used to develop themes from the interviews. The two researchers met periodically throughout data analysis to discuss and compare identified themes. Disagreements were resolved by discussion with the research team. This was compared to field notes collected during observation of ward rounds. After themes were finalized, they were mapped to the Theoretical Domains Framework (TDF)^15^ and the Capability, Opportunity, Motivation‐Behaviour (COM‐B) model.^16^ While distinct frameworks, the TDF and COM‐B model complement each other and the TDF domains further analyse the determinants of behaviour outlined in the COM‐B model. Results from surveys, interviews, focus groups and observation were compared using a triangulation design model.^17^ Analysis was performed using Microsoft Excel and NVivo 14.

## 3 RESULTS

### 3.1 Participant characteristics

Fifty‐eight healthcare professionals participated in surveys and 15 participated in an interview or focus group. The mean duration of the seven interviews was 32 min (range 27–38 min) and the mean duration of the three focus groups was 34 min (range 25–51 min). Most pharmacists, interns and junior residents had recent experience in more than one surgical specialty (including orthopaedic surgery, general surgery, plastic surgery, cardiothoracic surgery, vascular surgery, neurosurgery, urology and trauma surgery). Fifty‐one patients or carers participated in surveys and 13 participated in interviews or focus groups. The mean duration of the six interviews was 18 min (11–26 min) and the mean duration of the three focus groups was 24 min (range 21–28 min). Participant characteristics are summarized in Table 1. One investigator completed observation of surgical ward rounds with three surgical teams across two sites with eight ward round staff participants. The duration of observation was 6.5 h with 46 clinician–patient interactions and three clinician–carer interactions observed.

**TABLE 1:** Study participant characteristics.

Healthcare professional characteristics		
	Surveys (*n* = 58)	Interview or focus group (*n* = 15)
**Role**		
Pharmacists	15	4
Interns and residents	20	6
Registrars and consultants	23	5
**Age (years)**		
18–24	7	0
25–34	29	13
35+	22	2
**Sex**		
Male	21	6
Female	36	9
**Hospital** ^**a**^		
Hospital A (metropolitan tertiary hospital)	36	11
Hospital B/C (metropolitan secondary hospitals)	5	4
Hospital D/E (regional hospitals)	12	2
Other	9	N/A

Patient/carer characteristics		
	Surveys (*n* = 51)	Interview or focus group (*n* = 13)
**Role**		
Patient	34	9
Carer	17	4
**Age of patient (years)**		
65–74	19	2
75–84	21	5
85+	10	6
Missing	1	0
**Sex**		
Male	24	7
Female	27	6
**Hospital** ^**b**^		
Hospital A (metropolitan tertiary hospital)	23	8
Hospital B/C (metropolitan secondary hospitals)	8	5
Hospital D/E (regional hospitals)	7	1
Other	19	N/A
**Type of admission** ^**b**^		
Elective	27	9
Emergency	23	9
Missing	1	0
**Number of medications taken by patient**		
0–4	15	4
5–9	30	8
10+	6	1
**Admitting team** ^b^		
Orthopaedics	19	11
General surgery (including colorectal, upper gastrointestinal surgery and breast/endocrine surgery)	13	3
Other surgery (including plastic surgery, cardiothoracic, vascular, neurosurgery, urology)	23	5

^a^ Some participants worked at more than one hospital.

^b^ Some participants had multiple surgical admissions across different hospitals.

### 3.2 Survey results

**FIGURE 1:**
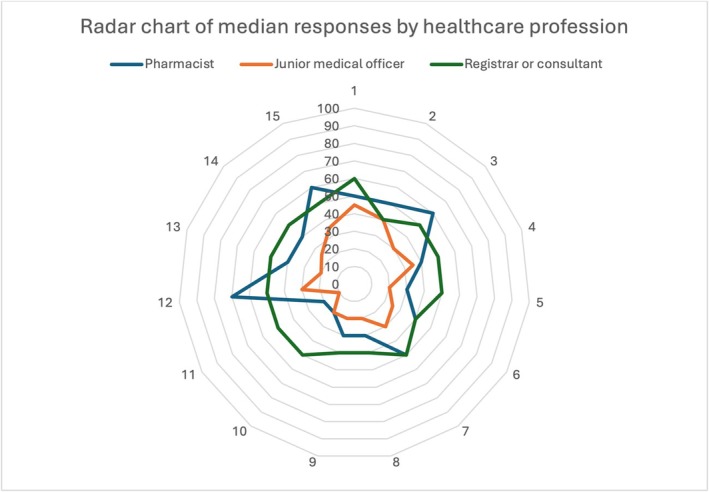
Radar chart of healthcare professionals' responses to the Deprescribing Self‐Efficacy Survey.^13^ Deprescribing under potentially impeding circumstances with participants rating of self‐efficacy on a scale of 0 (cannot do at all) to 100 (highly certain can do). 1. When I am concerned about adverse drug withdrawal events. 2. When I am concerned about exacerbations of the underlying condition the drug is being used to treat. 3. When disease‐specific clinical guidelines recommend the use of a medication. 4. When the medication is coupled to performance indicators. 5. When I receive little support from colleagues for stopping or reducing medications. 6. When I have too much work to do. 7. When I am concerned about damage to my provider–patient relationship. 8. When the patient is resistant to change. 9. When the patient's family/caregivers are resistant to change. 10. When there is no literature describing the effects of medication tapering or discontinuation. 11. When there is no guidance on how to taper or stop a medication. 12. When I am not the original prescriber of the medication. 13. When the medication was prescribed by a specialist. 14. When I am unsure why the medication was started originally. 15. When the medication is being used to treat an adverse effect of another medication.

Healthcare professional responses to the Deprescribing Self‐Efficacy Survey and the differences in responses between groups are summarized in Figure 1 and Supplemental Figure S1. Registrars (doctors undergoing specialist surgical training) and consultants were moderately confident deprescribing under all circumstances (median score 40–60 for all questions). Junior Medical Officers who have not commenced specialty training (JMOs) and pharmacists were less confident deprescribing (median score ≤30) when they received little support from colleagues, when patients and/or carers are resistant to change, and when there is no guidance on how to taper or stop a medication or the effects of this. JMOs were also less likely to deprescribe when the medication was prescribed by a specialist (median score 20).

**FIGURE 2:**
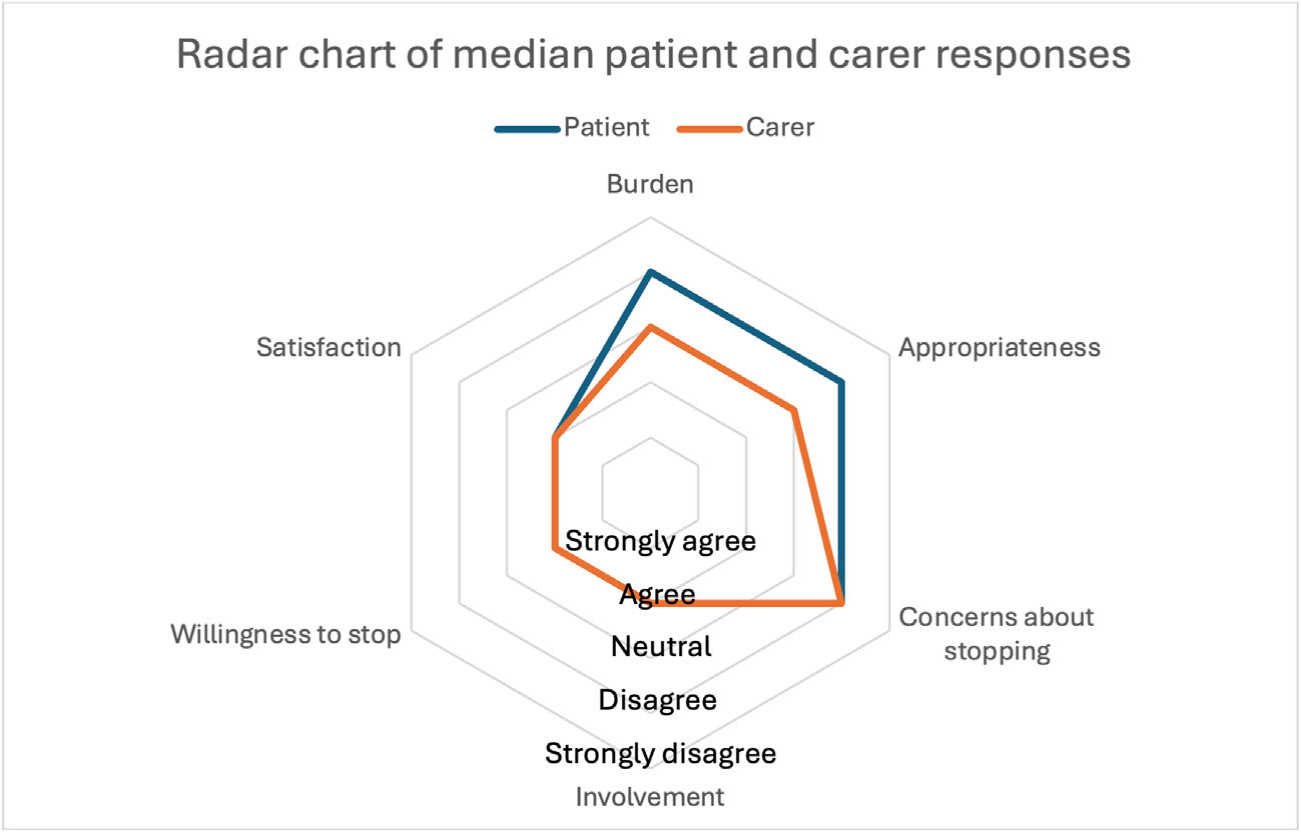
Radar chart of patient and carer responses to the revised Patients' Attitudes Towards Deprescribing questionnaire.^12^ Patient and carer question groups. Burden factor (5 questions): 1. Medicines are expensive, 2^a^. Medicines are inconvenient, 3. Taking a large number of medicines, 4. Medicines are a burden, 5. Taking too many medicines. Appropriateness factor (5 questions): 1. Taking medicines that are no longer needed, 2. Would like to try stopping medicines, 3. Would like to reduce the dose, 4. Medicines may not be working, 5. Medicines may be giving side effects. Concerns about stopping factor (5 questions): 1. Reluctance to stop long term medicines, 2^a^. Concerns about missing out on future benefits, 3. Stressed when medicines are changed, 4. Concern that doctors stopping medicine is giving up, 5. Previous bad experience with stopping medicine. Involvement factor (5 questions): 1^a^. Good understanding of medicines, 2. Knowledge of current medicines, 3. Would like to know as much as possible about medicines, 4. Would like to be involved in decision making about medicines, 5. Willingness to ask healthcare professionals if unsure of medicines. Willingness to stop (1 question): 1. Willingness to stop medicines if doctor said it was possible. Satisfaction (1 questions): 1. Satisfaction with current medicines. ^a^ Patient questions where there is no carer equivalent question.

Patient and carer responses to the rPATD questionnaire are summarized in Figure 2 and Supplemental Figures S2 and S3. Most (52.3%) patients disagreed or strongly disagreed that their medications were a burden or that they were inappropriate. There were mixed responses to questions regarding concerns about stopping. Most (87.5%) patients agreed or strongly agreed they wanted to be involved in decisions about their medications. In contrast, carers had mixed responses to questions regarding the burden and appropriateness of their care recipient's medications. Most (87%) carers disagreed, strongly disagreed or were neutral in responses to questions on concerns about stopping their care recipient's medications. Most (78%) carers agreed or strongly agreed that they wanted to be involved. While most patients (97%) and carers (71%) agreed or strongly agreed they were satisfied with their current medications, almost all (98%) also agreed or strongly agreed they would be willing to stop one of more regular medications if their doctor said it was possible.

### 3.3 Interview and observation results

Five themes were identified from the interviews, focus groups and observation of ward rounds. Themes and subthemes are summarized in Table 2, together with the corresponding TDF domain and COM‐B component. The subthemes were mapped to 11 out of the 14 domains.

**TABLE 2:** Themes and subthemes with interview quotes and observations together with the Theoretical Domains Framework (TDF) domain and Capability, Opportunity, Motivation‐Behaviour (COM‐B) component.

Deprescribing is not the priority		
**Deprescribing may be beneficial but is often not implemented in practice**	“I do not really go out of my way to look for medications that could be causing harm.” (Registrar 1) Observation: *deprescribing was not observed on surgical ward rounds*	Social influences (opportunity)
**Surgical priorities**	“Sometimes surgeons like do not really think, like, care about their medical issues. And just expect you or someone else to deal with them.” (JMO 4) Observation: *ward round discussions were focused on surgical management and the discharge plan*	Motivation and goals (motivation)
**Admission medications are often not correctly charted**	“In particularly the high turnover surgical wards, a patient does not even have their regular medicines written up until they go home.” (Pharmacist 1)	Social influences (opportunity)
**Limited time and high workload**	“They're busy people, so they do not want to take you on as a PhD student in a short stay.” (Patient 5) Observation: *in some teams, a very limited amount of time was available to review all patients before the surgical team was required in theatre*	Environmental context and resources (opportunity)

Medication review occurs in response to triggers		
**Time points**	“Truthfully, it was on discharge unless we were called so either discharge or pre‐op were probably your two major places. Pre‐op there's the anaesthetic considerations of withholding ACE inhibitors and diabetic medications but beyond that, mainly just on discharge to be honest.” (JMO 1) Observation: *surgical teams often document notes in retrospect and often do not access the electronic medical record or medication chart during ward rounds*	Environmental context and resources (opportunity)
**Reactive deprescribing**	“I think I'm happy deprescribing in situations where there is immediate danger to a patient so like stopping a benzodiazepine when a patient is drowsy or if someone is hypotensive, stopping their blood pressure medications.” (Registrar 3)	Beliefs about Consequences (motivation)
**The need for verbal prompts**	“What doctors need is someone external to the [surgical] team, tapping them on the shoulder to say that patient's been in for 3 days, no one's confirmed their medications.” (Consultant 1) Observation: *medication review did occur occasionally in response to patients asking about medications or symptoms but only specific medications were reviewed*	Behavioural regulation (capability)

Knowledge of deprescribing is limited		
**Conceptualization of deprescribing varies**	“I modify their use in a way, sometimes things are being used at the wrong time of day or antihypertensives, the other group, you know they might have hypertension and they are taking … they could change the time of day of some of the medications.” (JMO 5)	Knowledge (capability)
**Limited knowledge and confidence**	“Something the registrar would not address or they would not know.” (JMO 4)	Beliefs about capabilities (motivation)
**Education and guidelines**	“I think that all junior doctors would benefit from more knowledge about deprescribing and knowing what resources to go to, and I think pharmacists as well would also benefit from that too, especially newer early career pharmacists.” (Pharmacist 4)	Skills (capability)

Deprescribing is a team effort		
**Deprescribing is a shared responsibility**	“I think like it's everyone's responsibility. But then I think at the same time, my expertise probably means I should not be doing it on my own.” (JMO 3)	Social/professional role and identity (motivation)
**Seniority and support**	“Just having someone senior and experienced being able to say, look that's quite reasonable, I agree. I think that's probably the big thing.” (JMO 1) Observation: *surgical teams may defer to surgical liaison services for prescribing decisions when uncertain*	Beliefs about capabilities (motivation)

There is a need for trust, rapport and communication for deprescribing to occur		
**Trust and rapport**	‘I think it's about trust. I trust my GP and my cardiologist. If I trusted the doctors here then okay.’ (Patient 4)	Emotion (motivation)
**Simplified, clear communication is key**	“I do not want a long list of things. Because no‐one's going to read all of that. So just tell me what I need to know.” (Patient 9)	Memory, attention and decision processes (capability)
**Communication and involvement of the relevant parties**	“The communication is really vital and it's not just for the patient, it basically needs to be with the family as well so that they are all on board with the changes.” (Carer 2) Observation: *family members were infrequently present at the bedside during ward rounds*	Social influences (opportunity)

#### 3.3.1 Theme 1: Deprescribing is not the priority

##### Deprescribing may be beneficial but is often not implemented in practice

Most doctors and pharmacists identified that deprescribing may be beneficial for patients.

“Absolutely it should be done, on admission really. There's not many places along the health journey where people get deprescribed … and I think hospital admissions are a perfect place for them.” (JMO 1)

While surgical doctors identified there was a benefit to deprescribing, they expressed they rarely deprescribed. This was consistent with the observation of surgical ward rounds where deprescribing was not observed.

##### Surgical priorities

Surgical doctors did not consider deprescribing or medication management a priority. Competing clinical interests took priority over medication review and deprescribing.

“My job is more communicating what we're doing in terms of their operation and their postoperative rehabilitation and the surgical side of things.” (Registrar 1)

Some patients and carers wanted their medications reviewed in hospital. However, some patients and carers were focused on the surgery and did not express that deprescribing was a priority in the context of their surgical admission.

“I was just worried about Dad because it's a pretty big operation for an 86 year old, so my focus was probably not so much on his medications.” (Carer 3)

##### Admission medications are often not correctly charted

For some patients, correct charting of usual medications was a priority. However, both doctors and pharmacists reported medications were often charted incorrectly or not charted.

“20%, there would be 0 discrepancies at all. It's probably like 60% where there was like maybe one medication discrepancy during the admission.” (JMO 3)

Surgical healthcare professionals identified that patients often were not aware of their usual medications and the potential inaccuracy of charted medications in hospital made deprescribing challenging.

“[some have a] complete lack of knowledge of what they're on and why.” (Consultant 1)

##### Time and workload

All healthcare professionals listed time or workload as barriers to medication review. There was variation between different organizations and surgical specialties in terms of workload and support.

“A lot of pharmacists cover, you know, maybe two wards when ideally they'd be covering one, so you know, there could be a 60 sort of patient load rather than ideally more like a 30, so that definitely prevents regular medication review from happening.” (Pharmacist 4)

Patients and carers were also aware that healthcare professionals had competing priorities and, in some cases, this limited their willingness to ask about medications.

#### 3.3.2 Theme 2: Medication review occurs in response to triggers

##### Time points

Surgical healthcare professionals identified specific times where they believed medication review should occur such as preadmission clinic, on admission, on ward rounds and discharge. However, surgical doctors and pharmacists reported that this did not always occur in practice and medication review often did not occur during ward rounds. In some cases, the first medication review occurred at discharge.

“It's … like getting to the discharge and getting a discharge reconciliation and then querying a medication, and then you're like, I don't have time to speak to all these teams, the patient's going.” (JMO 3)

##### Reactive deprescribing

Surgical healthcare professionals reported they were more likely to review medications and deprescribe in response to adverse effects of medications.

“If they're hyponatraemic then I might recommend that their diuretic be stopped, or if they're in an SSRI for example, then we'll look at why they were on that in the first place.” (Pharmacist 3)

In some cases, these deprescribing decisions were not intentional but rather occurred due to infrequent review of medications.

“I think that is a point where you realize you've inadvertently in hospital deprescribed. Like you've accidentally withheld … one of the blood pressure medications and their blood pressure's been perfect throughout. So you're like … I guess I've just deprescribed something.” (JMO 4)

However, healthcare professionals were less likely to deprescribe where there was no adverse event or where they were concerned that changes could result in an adverse event.

##### The need for verbal prompts

Prompts from patients or other healthcare professionals were viewed as beneficial. Most surgical doctors often only reviewed medications in response to prompts.

“Not without prompting to be honest, either prompting from like a consulting service or the pharmacist or the patient themselves.” (JMO 4)

Electronic prompts were viewed as fatiguing for healthcare professionals.

“All the reminders on the electronic medical record, just ignore them after a while and they just stopped working.” (Consultant 1)

#### 3.3.3 Theme 3: Knowledge about deprescribing is limited

##### Conceptualization of deprescribing varies

While pharmacists were able to provide an accurate definition of polypharmacy and deprescribing, some surgical JMOs were less familiar with these terms. The JMOs interchangeably used the terms “withholding” and “deprescribing” medications.

“Deprescribing has been because they need surgery or they're kind of bleeding or something so it's contraindicated whereas I haven't seen much deprescribing that's just because it will benefit the patient in future.” (JMO 3)

##### Limited knowledge and confidence

Consistent with the survey results, JMOs felt they lacked knowledge and confidence in deprescribing. Surgical registrars and consultants were also perceived to have limited knowledge about deprescribing. Some pharmacists also felt they lacked confidence due to a lack of knowledge.

“It might have a shakier recommendation, and sometimes I feel it is appropriate to go to more of someone with that specialist deprescribing knowledge.” (Pharmacist 1)

Surgical doctors were focused on analgesics, antibiotics and blood thinners when reviewing a patient's medications. This was consistent with observations on ward rounds. Surgical healthcare professionals had differing levels of confidence with reviewing medications depending on their specialty and previous experiences. Most surgical doctors felt they did not have the knowledge to deprescribe “medical drugs” such as anticholinergic and sedative medications while level of confidence varied between pharmacists.

“Thyroid stuff, arrhythmic stuff, off the top of my head, there's a lot that I won't touch. Blood thinners, short term yes, long term no.” (Registrar 1)

##### Education and guidelines

Education of JMOs on medication review and deprescribing was viewed as beneficial. Surgical registrars felt that education was unlikely to be of benefit due to other priorities.

“Education is always really helpful, but you know whether we actually have the time or cognitive space … I think for registrars, I don't think there's really any point because we have gone down a different path.” (Registrar 2)

However, education for JMOs and junior pharmacists was identified as being of benefit. Knowledge of deprescribing guidelines and resources was limited amongst surgical JMOs. The importance of knowledge about resources was identified.

“I think just ensuring that everybody knows like a few top go‐to resources to refer to.” (Pharmacist 4)

#### 3.3.4 Theme 4: Deprescribing is a team effort

##### Deprescribing is a shared responsibility

Deprescribing was viewed as a shared responsibility by healthcare professionals.

“I think it's shared responsibility. I don't feel it should fall onto one particular specialty, subspecialty or health professional. I think it should be a collaborative approach and it should be shared.” (Pharmacist 1)

In contrast, patients often felt that it was the doctor's responsibility to manage medication changes and review medications. Additionally, several patients were not aware that pharmacists reviewed medications and viewed their role as supplying medications.

“It should be the doctor changing the medications. The doctor can order the medication with the pharmacist.” (Carer 2)

##### Seniority and support

JMOs were responsible for implementing most medication changes. However, they did not feel confident in making changes independently. They sought support from pharmacists, surgical registrars and surgical liaison services such as orthogeriatrics, to action changes. While surgical doctors were willing to change medications in the short term, to action medication changes for “medical drugs” long term, they preferred to seek support from medical teams. There were different existing support systems in place and this varied according to the location and specialty. There were also differences between the amount of support required by surgical healthcare professionals to implement changes, although JMOs expressed they generally would not action medication changes without senior support unless there was an immediate risk to the patient. Patients also considered the seniority of the doctor making the recommendations.

“It was only coming from the top people in here that I listened to.” (Patient 2)

#### 3.3.5 Theme 5: There is a need for trust, rapport and communication for deprescribing to occur

##### Trust and rapport

Rapport within the healthcare team was important in order to action deprescribing recommendations.

“I think to make a deprescribing decision, you need to have the relationship with the registrar or even the consultant … after you've built a rapport with them, they usually just accept your recommendation.” (Pharmacist 1)

Where there was a surgical liaison service available, there was often established rapport with that service and surgical doctors often deferred to the service for medication review. For some patients, developing trust and rapport with the healthcare team was essential. Other patients expressed automatic trust in the healthcare team. Length of stay was viewed as a barrier to medication review and deprescribing by healthcare professionals due to the limited time to build rapport.

##### Simplified, clear communication is key

Communication of medication changes was viewed by patients as important, particularly at the time of discharge. Most patients had limited knowledge regarding medication changes in hospital. Some patients expressed a preference for face‐to‐face verbal communication by a doctor in addition to written communication. Other patients were accepting of clear written communication only. Patients expressed the need to avoid jargon and the need for simple verbal communication of pertinent points including the name of the medication, indication, adverse effects and duration.

“Discuss what the different medications would do with the different side effects, the length of time you would have to take them, talk about them.” (Patient 1)

##### Communication and involvement of the relevant parties

Patients and carers expressed varying degrees of a need for communication and involvement in discussions. Some patients also expressed that communication with their general practitioner regarding ongoing changes was important while all surgical healthcare professionals rarely contacted general practitioners.

“Just as long as they communicate with my GP to tell them why it was changed and see, that doesn't get done.” (Patient 4)

Surgical doctors reported often communicating medication changes in the discharge summary rather than by verbal discussion. The availability of general practitioners and carers throughout the admission was viewed as a barrier to communication by surgical healthcare professionals. The early timing of most of the observed surgical ward rounds may have limited communication with carers during the ward round.

## 4 DISCUSSION

This study suggested surgical doctors and pharmacists had low to moderate levels of confidence in deprescribing and there were gaps in deprescribing capability. Previous studies, which mostly included primary care and hospital physicians, suggested most physicians reported general confidence in their ability to deprescribe and had reasonable knowledge about deprescribing.^18^, ^19^, ^20^ In contrast, deprescribing was conceptualized differently in this study with modifying medication timing or withholding a medication preoperatively being considered deprescribing by some doctors. This presents a unique challenge when addressing the capability of healthcare professionals in deprescribing in surgical settings. A lack of knowledge has been identified previously as a barrier to deprescribing.^21^, ^22^ In addressing the capability of healthcare professionals, the importance of education and guidelines is highlighted in this study and previous studies.^23^, ^24^ The importance of ensuring this information is relevant and can be easily understood by healthcare professionals and patients or carers has also been highlighted in this study and other studies.^25^ Additionally, the need for prompts to review medications, particularly verbal prompts by patients, carers or other healthcare professionals, in changing behaviour was identified.

Motivation and confidence to deprescribe was low amongst surgical healthcare professionals. Previous studies have suggested that deprescribing may not be a priority, particularly when there are no clinically obvious issues.^21^ Standards of practice in surgery do not incorporate deprescribing.^26^ Recent changes in policy, including requirements for medication review of high‐risk patients for hospital accreditation, may increase motivation.^27^ Like other studies, we found that deprescribing often occurs in response to triggers.^22^, ^28^ The importance of teamwork as well as involvement of patients and/or carers has been explored in previous studies.^29^ Deprescribing was viewed as a shared responsibility. Overall, it was not viewed as part of the role of a surgeon or surgical registrar while JMOs lacked the confidence to deprescribe. Surgical doctors felt supported by pharmacists and surgical liaison services where this was available. Similar to previous studies, patients were willing to consider deprescribing if their doctor said it was possible.^5^, ^30^ Additionally, as reported in the community setting,^31^ there was a need for trust and rapport with the hospital team to successfully deprescribe.

Healthcare professionals were able to identify time points in the hospital setting where there were potential opportunities for medication review and deprescribing. However, consistent with findings in other settings,^31^ time constraints and high workload prevent this occurring. While deprescribing was viewed as beneficial, surgical doctors rarely implement it in practice. Incorrect charting of medications in the hospital setting is common,^32^ and this was identified as a barrier that needed to be addressed before deprescribing could be considered by healthcare professionals and patients.

While clinical pharmacology and prescribing education for medical students have improved with the Australian/New Zealand adaptation of the UK Prescribing Safety Assessment,^33^ future interventions should focus on providing education and resources for practising surgical JMOs and pharmacists. Given the high workload, these resources should be easily accessible and succinct. In addition to further education, there is also a need for senior support in deprescribing as surgical registrars and surgeons are unlikely to deprescribe even with further education. Surgical healthcare professionals felt that deprescribing may be beneficial but reported low motivation to deprescribe due to other priorities. To address this as well as identify opportunities for deprescribing, proactive medication reconciliation and deprescribing with clear communication and verbal prompting should be integrated into existing surgical liaison services and pharmacist reviews. Additionally, patients and carers should be encouraged to maintain an accurate home medication list and ask about their medications in hospital.

While a strength of the study was that it was a multisite study conducted across five hospitals, a large number of participants were recruited from orthopaedics and Hospital A and this may limit the generalizability of the results. Additionally, there was variation between organizations and individual surgical teams within the same local health districts and it is likely that interventions will need to be tailored to individual surgical teams. The interviewer and observer was a geriatrician working at Hospital A and this may introduce bias. However, we used validated surveys and a semi‐structured interview guide. Additionally, a second researcher, who was not involved in study design or data collection, independently coded the interview transcripts.

This study suggested that while deprescribing was identified as potentially beneficial, surgical doctors are unlikely to proactively review medications and deprescribe medications. Motivation to deprescribe was low and it was not seen as a priority. This could be addressed through policy requirements, better evidence on the clinical impact of deprescribing in surgical patients, use of triggers and patient education. Support for patients, carers and healthcare practitioners to enable accurate documentation of medication history on admission is an essential prerequisite that must be addressed prior to medication review and deprescribing interventions. A collaborative patient‐centred approach with involvement from geriatricians, clinical pharmacologists, other physicians and/or pharmacists may improve prescribing in older surgical inpatients. Knowledge and confidence in deprescribing amongst surgical healthcare professionals was low, particularly amongst junior doctors. Education of JMOs and pharmacists may assist with bridging the gaps in knowledge and capability.

## AUTHOR CONTRIBUTIONS

BL, JT, KF, DG and SH designed the study. BL collected the data. BL and AL analysed the data. Data analysis was also discussed with JT, KF, DG and SH. BL drafted the initial manuscript, all authors revised the manuscript and approved the final version for submission.

## CONFLICT OF INTEREST STATEMENT

The authors state that there are no conflicts of interest to declare.

## Supporting information


**Figure S1** Bar chart of healthcare professionals' responses to the Deprescribing Self‐Efficacy Survey.
**Figure S2** Bar chart of patient responses to the revised Patients' Attitudes Towards Deprescribing questionnaire.
**Figure S3:** Bar chart of carer responses to the revised Patients' Attitudes Towards Deprescribing questionnaire.

## Data Availability

Data are available in the article supplementary material. Additional data are available on request due to privacy/ethical restrictions.
