# Supplementary material for: Patient, carer and healthcare professional perspectives on deprescribing in surgical wards: A mixed methods study
Source: Br J Clin Pharmacol. 2025 May 12;91(9):2684–95. doi: 10.1002/bcp.70088 (PMC12381604; doi:10.1002/bcp.70088)
Supplement: Supplementary file 1 — Figure S1 Bar chart of healthcare professionals' responses to the Deprescribing Self‐Efficacy Survey. Figure S2 Bar chart of patient responses to the revised Patients' Attitudes Towards Deprescribing questionnaire. Figure S3: Bar chart of carer responses to the revised Patients' Attitudes Towards Deprescribing questionnaire. [file BCP-91-2684-s001.docx]

**Supplemental Text S1**

**HEALTHCARE PROFESSIONAL SURVEY QUESTIONS**

DEPRESCRIBING UNDER POTENTIALLY IMPEDING CIRCUMSTANCES

What is your profession currently?

- Pharmacist- intern (less than 1 years’ experience)
- Pharmacist- 1-5 years’ experience
- Pharmacist- More than 5 years’ experience
- Doctor- intern
- Doctor- resident
- Doctor- registrar
- Doctor- consultant

What is your age?

- 18-24
- 25-34
- 35-44
- 45-54
- 55-64
- >65

What is your sex?

- Male
- Female
- Other

Which hospital do you work at?

- Royal North Shore Hospital
- Hornsby Hospital
- Ryde Hospital
- Gosford Hospital
- Wyong Hospital
- Other (please specify): _____________

This survey asks about deprescribing under potentially impeding circumstances. Deprescribing is the supervised process of dose reduction or stopping a medication that might be causing harm, or no longer be of benefit.

A number of situations are described below which can make it difficult to deprescribe medications in the elderly. Please rate how certain you are right now that you can deprescribe medications in the elderly by recording a number, 0 to 100 using the scale given below:

1. When I am concerned about adverse drug withdrawal events

- 0 (cannot do at all)
- 10
- 20
- 30
- 40
- 50 (moderately certain can do)
- 60
- 70
- 80
- 90
- 100 (Highly certain can do)

2. When I am concerned about exacerbations of the underlying condition the drug is being used to treat

- 0 (cannot do at all)
- 10
- 20
- 30
- 40
- 50 (moderately certain can do)
- 60
- 70
- 80
- 90
- 100 (Highly certain can do)

3. When disease-specific clinical guidelines recommend the use of a medication

- 0 (cannot do at all)
- 10
- 20
- 30
- 40
- 50 (moderately certain can do)
- 60
- 70
- 80
- 90
- 100 (Highly certain can do)

4. When the medication is coupled to performance indicators

- 0 (cannot do at all)
- 10
- 20
- 30
- 40
- 50 (moderately certain can do)
- 60
- 70
- 80
- 90
- 100 (Highly certain can do)

5. When I receive little support from colleagues for stopping or reducing medications

- 0 (cannot do at all)
- 10
- 20
- 30
- 40
- 50 (moderately certain can do)
- 60
- 70
- 80
- 90
- 100 (Highly certain can do)

6. When I have too much work to do

- 0 (cannot do at all)
- 10
- 20
- 30
- 40
- 50 (moderately certain can do)
- 60
- 70
- 80
- 90
- 100 (Highly certain can do)

7. When I am concerned about damage to my provider-patient relationship

- 0 (cannot do at all)
- 10
- 20
- 30
- 40
- 50 (moderately certain can do)
- 60
- 70
- 80
- 90
- 100 (Highly certain can do)

8. When the patient is resistant to change

- 0 (cannot do at all)
- 10
- 20
- 30
- 40
- 50 (moderately certain can do)
- 60
- 70
- 80
- 90
- 100 (Highly certain can do)

9. When the patient’s family/caregivers are resistant to change

- 0 (cannot do at all)
- 10
- 20
- 30
- 40
- 50 (moderately certain can do)
- 60
- 70
- 80
- 90
- 100 (Highly certain can do)

10. When there is no literature describing the effects of medication tapering or discontinuation

- 0 (cannot do at all)
- 10
- 20
- 30
- 40
- 50 (moderately certain can do)
- 60
- 70
- 80
- 90
- 100 (Highly certain can do)

11. When there is no guidance on how to taper or stop a medication

- 0 (cannot do at all)
- 10
- 20
- 30
- 40
- 50 (moderately certain can do)
- 60
- 70
- 80
- 90
- 100 (Highly certain can do)

12. When I am not the original prescriber of the medication

- 0 (cannot do at all)
- 10
- 20
- 30
- 40
- 50 (moderately certain can do)
- 60
- 70
- 80
- 90
- 100 (Highly certain can do)

13. When the medication was prescribed by a specialist

- 0 (cannot do at all)
- 10
- 20
- 30
- 40
- 50 (moderately certain can do)
- 60
- 70
- 80
- 90
- 100 (Highly certain can do)

14. When I am unsure why the medication was started originally

- 0 (cannot do at all)
- 10
- 20
- 30
- 40
- 50 (moderately certain can do)
- 60
- 70
- 80
- 90
- 100 (Highly certain can do)

15. When the medication is being used to treat an adverse effect of another medication

- 0 (cannot do at all)
- 10
- 20
- 30
- 40
- 50 (moderately certain can do)
- 60
- 70
- 80
- 90
- 100 (Highly certain can do)

**PATIENT/CARER SURVEY QUESTIONS**

INVESTIGATION INTO THE BELIEFS OF OLDER ADULTS ABOUT MEDICINES QUESTIONNAIRE

Are you a patient or carer?

- Patient
- Carer

Age of patient at time of hospital admission

- Less than 65
- 65-74
- 75-84
- 85+

Sex (please circle)

- Male
- Female
- Other

Which hospital were you (or the person you are caring for) admitted at (please circle)?

- Royal North Shore Hospital
- Hornsby Hospital
- Ryde Hospital
- Gosford Hospital
- Wyong Hospital
- Other (please specify): _____________

What year were you (or the person you are caring for) admitted?

- 2022
- 2023
- 2024

What was the admitting surgical team? ________________

Was this an elective or emergency admission (please circle)?

- Elective
- Emergency

Was surgery performed during the surgical admission (please circle)?

- Yes
- No

How long did you (or the person you are caring for) stay in hospital (please circle)?

- Less than 2 days
- 2-7 days
- 1-2 weeks
- 3-4 weeks
- >1 month

Approximately how many medications do you (or the person you are caring for) take (please circle)?

- 0-4
- 5-9
- 10+

**FOR PATIENTS** (Investigation into the beliefs of older adults about medicines questionnaire)

There are no right or wrong answers, please select the box to indicate how strongly you agree with each of the following statements. If there are any questions that you cannot answer, or feel that it doesn’t apply to you, please skip it and move to the next question.

For the questions that ask about your doctor, please think of the doctor that prescribes the most (if not all) of your medicines.

Overall, I am satisfied with my current medicines (check box)

- strongly agree
- agree
- unsure
- disagree
- strongly disagree

I like to be involved in making decisions about my medicines with my doctors

- strongly agree
- agree
- unsure
- disagree
- strongly disagree

I have a good understanding of the reasons I was prescribed each of my medicines

- strongly agree
- agree
- unsure
- disagree
- strongly disagree

I like to know as much as possible about my medicines

- strongly agree
- agree
- unsure
- disagree
- strongly disagree

I always ask my doctor, pharmacist or other health care professional if there is something I don’t understand about my medicines

- strongly agree
- agree
- unsure
- disagree
- strongly disagree

I know exactly what medicines I am currently taking, and/or I keep an up to date list of my medicines

- strongly agree
- agree
- unsure
- disagree
- strongly disagree

If my doctor said it was possible I would be willing to stop one or more of my regular medicines

- strongly agree
- agree
- unsure
- disagree
- strongly disagree

I feel that I am taking a large number of medicines

- strongly agree
- agree
- unsure
- disagree
- strongly disagree

Taking my medicines every day is very inconvenient

- strongly agree
- agree
- unsure
- disagree
- strongly disagree

I spend a lot of money on my medicines

- strongly agree
- agree
- unsure
- disagree
- strongly disagree

Sometimes I think I take too many medicines

- strongly agree
- agree
- unsure
- disagree
- strongly disagree

I feel that my medicines are a burden to me

- strongly agree
- agree
- unsure
- disagree
- strongly disagree

I would like to try stopping one of my medicines to see how I feel without it

- strongly agree
- agree
- unsure
- disagree
- strongly disagree

I would like my doctor to reduce the dose of one or more of my medicines

- strongly agree
- agree
- unsure
- disagree
- strongly disagree

I feel that I may be taking one or more medicines that I no longer need

- strongly agree
- agree
- unsure
- disagree
- strongly disagree

I believe one or more of my medicines may be currently giving me side effects

- strongly agree
- agree
- unsure
- disagree
- strongly disagree

I think one or more of my medicines may not be working

- strongly agree
- agree
- unsure
- disagree
- strongly disagree

I have had a bad experience when stopping a medicine before

- strongly agree
- agree
- unsure
- disagree
- strongly disagree

I would be reluctant to stop a medicine that I had been taking for a long time

- strongly agree
- agree
- unsure
- disagree
- strongly disagree

If one of my medicines was stopped I would be worried about missing out on future benefits

- strongly agree
- agree
- unsure
- disagree
- strongly disagree

I get stressed whenever changes are made to my medicines

- strongly agree
- agree
- unsure
- disagree
- strongly disagree

If my doctor recommended stopping a medicine I would feel that he/she was giving up on me

- strongly agree
- agree
- unsure
- disagree
- strongly disagree

**FOR CARERS** (Investigation into the beliefs of carers about medicines questionnaire)

There are no right or wrong answers, please select the box to indicate how strongly you agree with each of the following statements. If there are any questions that you cannot answer, or feel that it doesn’t apply to you, please skip it and move to the next question.

For the questions that ask about your care recipient’s doctor, please think of the doctor that prescribes the most (if not all) of their medicines

Overall, I am satisfied with my care recipient’s current medicines

- strongly agree
- agree
- unsure
- disagree
- strongly disagree

I like to be involved in making decisions about my care recipient’s medicines with their doctors

- strongly agree
- agree
- unsure
- disagree
- strongly disagree

I like to know as much as possible about my care recipient’s medicines

- strongly agree
- agree
- unsure
- disagree
- strongly disagree

I always ask the doctor, pharmacist or other health care professional if there is something I don’t understand about my care recipient’s medicines

- strongly agree
- agree
- unsure
- disagree
- strongly disagree

I know exactly what medicines the person that I care for is currently taking and/or I have an up to date list of their medicines

- strongly agree
- agree
- unsure
- disagree
- strongly disagree

If their doctor said it was possible I would be willing to stop one or more of my care recipient’s medicines

- strongly agree
- agree
- unsure
- disagree
- strongly disagree

I feel that the person I care for is taking a large number of medicines

- strongly agree
- agree
- unsure
- disagree
- strongly disagree

My care recipient’s medicines are quite expensive

- strongly agree
- agree
- unsure
- disagree
- strongly disagree

Sometimes I think the person I care for takes too many medicines

- strongly agree
- agree
- unsure
- disagree
- strongly disagree

I feel that my care recipient’s medicines are a burden to them

- strongly agree
- agree
- unsure
- disagree
- strongly disagree

I would like the doctor to try stopping one of my care recipient’s medicines to see how they feel without it

- strongly agree
- agree
- unsure
- disagree
- strongly disagree

I would like the doctor to reduce the dose of one or more of my care recipient’s medicines

- strongly agree
- agree
- unsure
- disagree
- strongly disagree

I feel that the person that I care for may be taking one or more medicines that they no longer need

- strongly agree
- agree
- unsure
- disagree
- strongly disagree

I believe one or more of my care recipient’s medicines may be currently giving them side effects

- strongly agree
- agree
- unsure
- disagree
- strongly disagree

I think one or more of my care recipient’s medicines may not be working

- strongly agree
- agree
- unsure
- disagree
- strongly disagree

The person that I care for has had a bad experience when stopping a medicine before

- strongly agree
- agree
- unsure
- disagree
- strongly disagree

I would be reluctant to stop one of my care recipient’s medicines that they had been taking for a long time

- strongly agree
- agree
- unsure
- disagree
- strongly disagree

I get stressed whenever changes are made to my care recipient’s medicines

- strongly agree
- agree
- unsure
- disagree
- strongly disagree

I feel that if I agreed to stopping one of my care recipient’s medicines then this is giving up on them

- strongly agree
- agree
- unsure
- disagree
- strongly disagree

**SEMI-STRUCTURED INTERVIEW/FOCUS GROUP QUESTION GUIDE (healthcare professionals)**

Preferred name, sex, age, role

Knowledge and practice

Can you describe your understanding of polypharmacy and deprescribing?

When do you review a patient’s medication chart and make medication changes? Do you review a patient’s medications on your ward round?

What do you do as part of a medication review for surgical inpatients? Prompt: do you consciously practice deprescribing?

Do you think deprescribing should be done in older surgical patients? Prompt: which type of medications do you think should be deprescribed in older surgical patients?

How often do you deprescribe for your surgical inpatients and what is your approach?

Do you think you have the appropriate knowledge to deprescribe medications? Why/why not?

How often do you communicate medication changes with surgical inpatients and/or their carers and their GPs?

Attitude

Whose responsibility do you think it is to perform a medication review and deprescribe for surgical inpatients?

Do you think polypharmacy is a problem in older surgical inpatients and why/why not?

Do you think deprescribing is important and why/why not?

In cases where you have deprescribed for surgical inpatients, what are your main reasons for deprescribing?

Barriers

Thinking about medication review in your surgical inpatients, what do you perceive are barriers that prevent you from completing / limit your ability to complete a medication review for them?

Are there any medications that are harder or easier to deprescribe in hospital for surgical inpatients? Prompt: what are they / can you please tell me about an experience where you found it easy/hard to deprescribe for a surgical inpatient?

What stops you from deprescribing in hospital medications for your surgical inpatients? Prompts: time etc

What are the main factors that prevent you from communicating with the surgical inpatients, carers and their GPs about their medications?

Facilitators

What do you think would help you with completing a medication review?

What would help you to reduce inappropriate polypharmacy or deprescribe medications in for your surgical inpatients? Prompt: tools, education

What would help you to communicate medication changes to surgical inpatients, carers and GPs?

Is there anything else you would like to add?

**SEMI-STRUCTURED INTERVIEW/FOCUS GROUP QUESTION GUIDE (patients/carers)**

Preferred name, sex, age, role

Knowledge and practice

How are you usually involved in decisions about your medications with your GP or specialists?

During your stay in hospital when you had surgery/were under a surgical team, were there changes that were made to your medications? If yes, can you tell me what changes were made and did you understand why these changes were made?

Do you remember anyone in hospital speaking to you about your medications? If yes, do you remember who and what was discussed?

Attitude

How did you feel about the changes made to your medications in hospital? How did you feel about doctors starting new medications? How did you feel about doctors stopping or reducing your usual medications?

What are your thoughts about doctors and pharmacists changing your medications in hospital? How would you feel if a doctor in hospital stopped or reduced one of your usual medications?

How did you feel about the conversations you had in hospital about your medications? Prompts: clarity of information, concerns addressed

Barriers

Thinking about your experience with medications while in hospital, is there anything that you have wanted to happen differently? Prompts: amount of information, clarity of information, people involved in discussions, communication with carers or GPs or others

Are there any medications that you would not want changed in hospital while you were having surgery and why? Prompts: perceived benefit

What would stop you from asking a doctor or pharmacist in hospital about your medications?

Facilitators

When you are in hospital for surgery, how can we help yourself and/or others understand the medication changes made? Prompts: written information, involvement in decisions, discussion with GP and carers

Are there any medications that you would want changed in hospital while you were having surgery and why? Prompts: side effects, cost

If medication changes are made in hospital, who do you want these decisions communicated to and how?

Is there anything else you would like to add?

Figure S1: Bar chart of healthcare professionals’ responses to the Deprescribing Self-Efficacy Survey

Figure S2: Bar chart of patient responses to the revised Patients' Attitudes Towards Deprescribing questionnaire

Burden factor

B1- I spend a lot of money on my medicines

B2- Taking my medicines every day is very inconvenient

B3- I feel that I am taking a large number of medicines

B4- I feel that my medicines are a burden to me

B5- Sometimes I think I take too many medicines

Appropriateness factor

A1- I feel that I may be taking one or more medicines that I no longer need

A2- I would like to try stopping one of my medicines to see how I feel without it

A3- I would like my doctor to reduce the dose of one or more of my medicines

A4- I think one or more of my medicines may not be working

A5- I believe one or more of my medicines may be currently giving me side effects

Concerns about stopping factor

C1- I would be reluctant to stop a medicine that I had been taking for a long time

C2- If one of my medicines was stopped I would be worried about missing out on future benefits

C3- I get stressed whenever changes are made to my medicines

C4- If my doctor recommended stopping a medicine I would feel that he/she was giving up on me

C5- I have had a bad experience when stopping a medicine before

I1- I have a good understanding of the reasons I was prescribed each of my medicines

Involvement factor

I2- I know exactly what medicines I am currently taking, and/or I keep an up to date list of my medicines

I3- I like to know as much as possible about my medicines

I4- I like to be involved in making decisions about my medicines with my doctors

I5- I always ask my doctor, pharmacist or other health care professional if there is something I don’t understand about my medicines

Global questions

G1- If my doctor said it was possible I would be willing to stop one or more of my regular medicines

G2- Overall, I am satisfied with my current medicines

Figure S3: Bar chart of carer responses to the revised Patients' Attitudes Towards Deprescribing questionnaire

Burden factor

B1-My care recipient’s medicines are quite expensive

B3- I feel that the person I care for is taking a large number of medicines

B4- I feel that my care recipient’s medicines are a burden to them

B5- Sometimes I think the person I care for takes too many medicines

Appropriateness factor

A1- I feel that the person that I care for may be taking one or more medicines that they no longer need

A2- I would like the doctor to try stopping one of my care recipient’s medicines to see how they feel without it

A3- I would like the doctor to reduce the dose of one or more of my care recipient’s medicines

A4- I think one or more of my care recipient’s medicines may not be working

A5- I believe one or more of my care recipient’s medicines may be currently giving them side effects

Concerns about stopping factor

C1- I would be reluctant to stop one of my care recipient’s medicines that they had been taking for a long time

C3- I get stressed whenever changes are made to my care recipient’s medicines

C4- I feel that if I agreed to stopping one of my care recipient’s medicines then this is giving up on them

C5- The person that I care for has had a bad experience when stopping a medicine before

Involvement factor

I2- I know exactly what medicines the person that I care for is currently taking and/or I have an up to date list of their medicines

I3- I like to know as much as possible about my care recipient’s medicines

I4- I like to be involved in making decisions about my care recipient’s medicines with their doctors

I5- I always ask the doctor, pharmacist or other health care professional if there is something I don’t understand about my care recipient’s medicines

Global questions

G1- If their doctor said it was possible I would be willing to stop one or more of my care recipient’s medicines

G2- Overall, I am satisfied with my care recipient’s current medicines
